# Large volume infusions of hydroxyethyl starch during cardiothoracic surgery may be associated with postoperative kidney injury: propensity-matched analysis

**DOI:** 10.1186/s13741-019-0125-z

**Published:** 2019-10-31

**Authors:** Wataru Matsunaga, Masamitsu Sanui, Yusuke Sasabuchi, Yasuma Kobayashi, Asuka Kitajima, Fumitaka Yanase, Yutaka Takisawa, Alan Kawarai Lefor

**Affiliations:** 1 Department of Anesthesiology and Critical Care Medicine, Jichi Medical University Saitama Medical Centre, 1-847 Amanumacho, Omiya-ku, Saitama City, Saitama 330-8503 Japan; 2 Data Science Centre, Jichi Medical University, 3311-1 Yakushiji, Shimotsuke, Tochigi 329-0498 Japan; 3 Department of Surgery, Jichi Medical University, 3311-1 Yakushiji, Shimotsuke City, Tochigi 329-0498 Japan

**Keywords:** Hydroxyethyl starch, Intraoperative, AKI, Kidney damage, Cardiothoracic surgery

## Abstract

**Background:**

The safety of intraoperative administration of hydroxyethyl starch (HES) has been debated. We hypothesized that intraoperative use of HES is associated with postoperative acute kidney injury (AKI) following cardiopulmonary bypass (CPB).

**Materials and methods:**

Patients who underwent cardiothoracic surgery using CPB between 2007 and 2014 were retrospectively reviewed. The incidence of AKI within 7 days after surgery, defined by the Kidney Disease Improving Global Outcome criteria, was compared for patients who did or did not receive 6% (70/0.5) or 6% (130/0.4) HES for anesthesia management before or after CPB. Multivariable logistic regression and propensity matching analysis were performed to examine whether use of HES is associated with postoperative AKI. Outcomes comparing patients receiving HES ≥ 1000 mL and < 1000 mL were also compared.

**Results:**

Data from 1976 patients were reviewed. All patients received 70/0.5 HES as a part of the priming solution for CPB. The incidence of postoperative AKI was 28.2% in patients who received HES and 26.0% in patients who did not (*p* = 0.33). In multivariable analysis, there was no correlation between the use of HES and the incidence of AKI (odds ratio 0.87, 95% CI 0.30–2.58, *p* = 0.81). Propensity matching showed that the incidence of AKI was not significantly different between 481 patients administered with HES and 962 patients (26.6% vs. 26.9%, *p* = 0.95) who did not receive HES for anesthesia management. However, peak creatinine levels, needed for renal replacement therapy, and in-hospital mortality were higher, and 28-day hospital-free days were lower in patients receiving HES ≥ 1000 mL than those receiving HES < 1000 mL (*p* < 0.05).

**Conclusions:**

Intraoperative use of HES was not associated with postoperative AKI following CPB. However, administration of large volumes of HES may be associated with kidney-related adverse clinical outcomes.

## Background

Maintaining intravascular volume by fluid administration is essential to maintain stable hemodynamic status, but optimal perioperative fluid management including the types and the amount of fluid infused is a matter of continuing debate. Colloid solutions are often used with the expectation of maintaining intravascular volume and improving perioperative outcomes compared to crystalloids (Thompson and Walton 1964). However, a substantial number of studies failed to show clinical benefits of colloids compared with crystalloids (Glover et al. 2014). Several studies showed that colloid solutions are harmful, including detrimental effects of hydroxyethyl starch (HES) on kidney function and overall prognosis. The deleterious effects of HES on kidney function have been well documented in critically ill (Myburgh et al. 2012) and septic patients (Perner et al. 2012) in the intensive care unit (ICU).

However, whether the intraoperative use of HES has a detrimental effect on postoperative kidney function is controversial. A meta-analysis of 19 randomized controlled trials (Gillies et al. 2014) comparing intraoperative use of HES with other fluid solutions concluded that HES is not associated with an increase in mortality during hospitalization or an increased need for renal replacement therapy (RRT). However, another systematic review suggested an increased risk of postoperative renal damage with the intraoperative use of HES (Ishihara 2014).

To clarify these discrepant results, we hypothesized that the intraoperative use of HES solution has a detrimental effect on postoperative kidney function after cardiopulmonary bypass (CPB). The association between intraoperative administration of HES, either 6% (130/0.4) or 6% (70/0.5) solution, and postoperative acute kidney injury (AKI) defined by the Kidney Disease Improving Global Outcome (KDIGO) criteria, was analyzed in patients who underwent CPB during cardiothoracic surgery.

## Materials and methods

Institutional Review Board (IRB) approval was obtained, and the need for informed consent was waived due to the nature of the study. All patients who underwent cardiothoracic surgery at Jichi University Saitama Medical Centre between 2007 and 2014 were included in this study. Patients under 18 years old, those who were undergoing RRT at the time of surgery, and those who did not have serum creatinine levels measured within 6 weeks before surgery were excluded. Patients who had missing data were excluded to maintain the quality of the study.

An electronic medical record maintains all patient information during the hospital stay (COSMOS, IBM Japan, Ltd., Japan,). Separate electronic medical record systems record perioperative information including information associated with anesthesia (ORSYS, Philips Electronics Japan, Ltd., Japan) and postoperative ICU information (PIMS, Philips Electronics Japan, Ltd., Japan). All patient information in the ORSYS and PIMS systems were extracted through Vi-Pros (DOWELL Co., Ltd., Japan) data mining software, except perioperative laboratory data and patient demographic information from COSMOS. Patient preoperative characteristics including age, gender, body mass index, emergency surgery, comorbidities (hypertension, diabetes mellitus, chronic obstructive pulmonary disease, and chronic heart failure), American Society of Anesthesiologists classification, preoperative use of angiotensin-converting-enzyme inhibitors/angiotensin receptor blockers, atrial fibrillation, and preoperative serum creatinine level within 6 weeks prior to surgery were collected as baseline information. Intraoperative data extracted included the type of surgery, operative time, cardiopulmonary bypass (CPB) time, maximum/total duration with a mean blood pressure lower than 65 mmHg, estimated blood loss, intraoperative urinary output, crystalloid and 6% (70/0.5) HES (Hespander®; Fresenius Kabi Japan Inc., Tokyo, Japan and Salinhes®; Fresenius Kabi Japan Inc., Tokyo, Japan) and 6% (130/0.4) HES (Voluven®; Fresenius Kabi Japan Inc., Tokyo, Japan) administered, and volume of blood transfused. The type and amount of transfusion was left to the anesthesiologists’ discretion. Postoperative parameters including maximum serum creatinine level within 7 days after surgery, need for RRT, ICU length of stay, hospital length of stay, and in-hospital mortality were extracted. Postoperatively, no patients received HES in the ICU, since avoiding HES administration is a part of our routine practice.

Patients were divided into two groups for review: a group who received any type of HES solution (the HES group) and others who did not receive HES (the control group) before or after CPB during surgery. Since 500 mL of Salinhes® was routinely used as a part of the priming solution for cardiopulmonary bypass, all study patients received an identical (i.e., 500 mL) volume of Salinhes®.

Multivariable logistic regression analysis was performed to estimate the effect of each type of HES solution (Hespander®, Salinhes®, and Voluven®) on the incidence of postoperative AKI using preoperative and intraoperative patient demographic parameters (as shown in Table 4). Each administration of 500 mL of Hespander®, Salinhes®, and Voluven® was used as an independent covariate to clarify each product’s effect on the incidence of postoperative AKI. To reduce the impact of selection bias, propensity score matching analysis was performed. The propensity score to receive HES before or after CPB was calculated using a logistic regression model using variables including preoperative and intraoperative patient demographic parameters (age, gender, body mass index, emergency surgery, comorbidities, American Society of Anesthesiologists classification, preoperative use of angiotensin-converting-enzyme inhibitors/angiotensin receptor blockers, preoperative serum creatinine levels, operative time, CPB time, estimated blood loss, intraoperative urinary output, crystalloid administrated, and volume of blood transfused). Each patient who received HES was matched to two patients who did not receive HES without replacement, using the method of Sekhon et al. with a caliper (Sekhon 2011).

The primary outcome of this study was the incidence of AKI within 7 days after surgery defined by the KDIGO criteria. Only creatinine criteria were applied due to the lack of data for postoperative urine volume. Secondary outcomes included the need for RRT, ICU length of stay, and hospital mortality. To examine the dose-dependent effects of HES, we divided all patients before propensity matching into two groups (those who received HES 1000 mL or more and those who received less than 1000 mL) and compared the incidence of AKI, the need for RRT, ICU length of stay, and hospital mortality between the two groups. The cost of the solutions (HES and crystalloids) administered to each patient during surgery was determined and compared between the two groups.

### Statistical analysis

Data are expressed as mean and standard deviation or median and 25% and 75% percentiles, as appropriate. The Mann-Whitney *U* test was used to compare continuous variables and the *χ*^2^ test was used to compare nominal variables. Multivariable logistic regression analysis was performed to estimate the effects of each type of HES solution (Hespander®, Salinhes®, and Voluven®) on the incidence of postoperative AKI using preoperative and intraoperative patient parameters (as listed above).

To compare patients who did or did not receive HES, standardized differences were calculated. Standardized differences less than 10% denote a negligible imbalance between the groups. Outcomes were compared using the Mann-Whitney *U* test for continuous variables and *χ*^2^ test for nominal variables. All statistical analyses were performed with EZR (Saitama Medical Centre, Jichi Medical University, Saitama, Japan), which is a graphical user interface for R (The R Foundation for Statistical Computing, Vienna, Austria) (Kanda 2013).

## Results

**Fig. 1:**
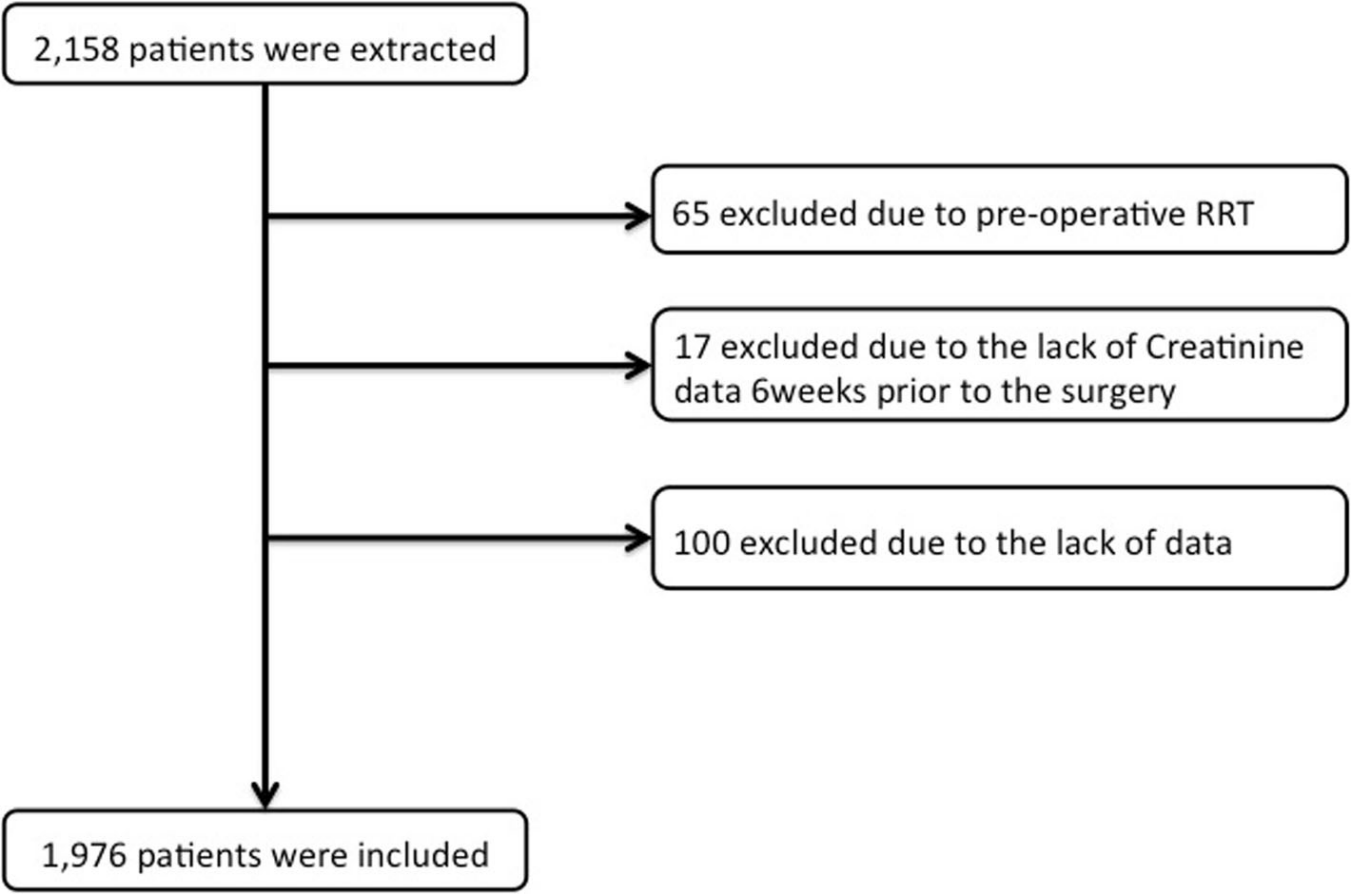
Study patient flow diagram

A total of 2158 patients who underwent cardiothoracic surgery from 2007 through 2014 were identified using the Vi-Pros® software. After applying exclusion criteria, 1976 patients were included in this study (Fig. 1) and 536 patients (27.1%) had HES administration with a mean volume of 719 mL and a median of 500 mL (500–1000 mL, as 25% and 75% percentiles) for anesthesia management. Preoperative patient characteristics are shown in Table 1. Patients who received HES were less likely to be male and less likely to be taking angiotensin-converting-enzyme inhibitor medications preoperatively. There was no difference in preoperative serum creatinine levels between the two groups.

**Table 1:** Demographic characteristics of study patients who did or did not receive HES before or after cardiopulmonary bypass

	HES	Control	Standardized difference (%)
Preoperative parameters			
Number of patients	536	1440	
Age (years)	65.8 ± 13.0	66.9 ± 12.0	− 1.46
Male gender	338 (63.1%)	782 (54.3%)	18.2
Body mass index (kg/m^2^)	22.7 ± 3.8	23.2 ± 3.8	7.10
Preoperative serum creatinine (mg/dL)	1.00 ± 0.81	0.92 ± 0.39	− 37.7
Emergency	202 (37.7%)	527 (36.6%)	2.33
Preoperative comorbidities			
Hypertension	328 (61.2%)	859 (59.7%)	3.22
Diabetes mellitus	79 (14.7%)	223 (15.5%)	− 2.91
Chronic obstructive pulmonary disease	12 (2.2%)	31 (2.2%)	2.00
Chronic heart failure	214 (39.9%)	598 (41.5%)	− 3.32
Atrial fibrillation	130 (24.3%)	320 (22.2%)	5.70
American Society of Anesthesiologists classification			
1	11 (2.1%)	24 (1.7%)	
2	329 (61.4%)	779 (54.1%)	
3	159 (30.0%)	523 (36.3%)	
4	31 (5.8%)	107 (7.4%)	
5	6 (1.1%)	7 (0.5%)	
Preoperative medications			
Angiotensin-converting-enzyme inhibitors	105 (19.6%)	210 (14.6%)	17.7
Angiotensin II receptor blockers	187 (34.9%)	466 (32.4%)	5.67
Intraoperative parameters			
HES (mL)	719 ± 422	–	
Hespander® (mL)	15 ± 105		
Salinhes® (mL)	655 ± 467		
Voluven® (mL)	49 ± 183		
Crystalloids (mL)	2549 ± 1611	2559 ± 1090	− 0.00025
Red blood cells (mL)	547 ± 1074	494 ± 763	0.0123
Fresh frozen plasma (mL)	486 ± 814	412 ± 592	0.0292
Platelet concentrates (mL)	119 ± 210	111 ± 190	0.0423
Autotransfusion (mL)	134 ± 292	76 ± 223	0.174
Estimated blood loss (mL)	807 ± 889	649 ± 605	0.0547
Urinary outputs (mL)	1412 ± 880	1338 ± 815	0.0207
Operative time (min)	372 ± 144	354 ± 110	0.220
Cardiopulmonary bypass time (min)	177 ± 79	168 ± 59	0.360
Longest interval of mABP < 65 (min)	70 ± 57	66 ± 53	0.279
Total mABP < 65 duration (min)	222 ± 124	211 ± 108	0.160
Operative procedure			
On-pump CABG	8 (1.5%)	20 (1.4%)	
Valve surgery	291 (54.3%)	734 (51.0%)	
Valve + CABG	36 (6.7%)	140 (9.7%)	
Major aortic surgery	172 (32.1%)	461 (32.0%)	
Other	29 (5.4%)	85 (5.9%)	

Data are expressed as means with standard deviation or percentages

*HES* hydroxyethyl starch, *mABP* mean arterial blood pressure, *CABG* coronary artery bypass graft

Outcomes are shown in Tables 2 and 3. The postoperative incidence of AKI was not different between the groups (28.2% in the HES group and 26.0% in the control group, *p* = 0.33). The need for RRT and hospital mortality was similar in both groups, while the ICU length of stay was longer in the HES group.

**Table 2:** Outcomes of patients who did or did not receive HES before or after cardiopulmonary bypass

	HES (n = 536)	Control (n = 1440)	*p* value
AKI incidents	151 (28.2%)	374 (26.0%)	0.33
AKI stage			
1	93 (17.4%)	229 (15.9%)	
2	20 (3.7%)	53 (3.7%)	
3	38 (7.1%)	92 (6.4%)	
Peak serum creatinine (mg/dL)	0.96 (0.77–1.27)	0.93 (0.71–1.35)	0.28
Need for RRT	30 (5.6%)	74 (5.1%)	0.73
ICU length of stay (days)	3 (3–5)	3 (3–4)	0.0079
28-day hospital free days (days)	11 (4–14)	12 (6–15)	0.060
Hospital mortality	22 (4.1%)	38 (2.6%)	0.10

Data are expressed as medians with 25th to 75th percentiles or percentages

*HES* hydroxyethyl starch, *AKI* acute kidney injury, *RRT* renal replacement therapy, *ICU* intensive care unit

**Table 3:** Outcomes of patients who received HES ≥ 1000mL or < 1000 mL

	HES ≥ 1000 mL	HES < 1000 mL	*p* value
Number of patients	179	357	
AKI incidents	57 (31.8%)	94 (26.3%)	0.22
AKI stage			
1	35 (19.6%)	58 (16.2%)	
2	3 (1.7%)	17 (4.8%)	
3	19 (10.6%)	19 (5.3%)	
Peak serum creatinine (mg/dL)	1.01 (0.81–1.33)	0.94 (0.75–1.25)	0.038
RRT requirement	16 (8.9%)	14 (3.9%)	0.017
ICU length of stay (days)	4 (3–5)	3 (3–5)	0.27
28-day hospital free days (days)	10 (0–14)	12 (5–14)	0.032
Hospital mortality	13 (7.3%)	9 (2.5%)	0.017

Data are expressed as medians with 25th to 75th percentiles or percentages

*HES* hydroxyethyl starch, *AKI* acute kidney injury, *RRT* renal replacement therapy, *ICU* intensive care unit

Multivariable analysis revealed that atrial fibrillation, body mass index, estimated blood loss (intervals of 100 mL), and FFP transfused (by units, 120 mL) were significantly associated with the incidence of postoperative AKI (Table 4). There was no correlation between the use of any of the three HES solutions studied and the incidence of AKI (Hespander®, odds ratio 1.94, 95% CI 0.43–8.72, *p* = 0.39; Salinhes®, odds ratio 0.74, 95% CI 0.02–35.7, *p* = 0.88; Voluven®, odds ratio 0.87, 95% CI 0.30–2.58, *p* = 0.81, respectively).

**Table 4:** Preoperative and intraoperative factors associated with postoperative acute kidney injury by multivariable logistic regression analysis

	Odds ratio (95% confidence interval)	*p* value
Age	1.04 (0.99–1.10)	0.13
Male gender	2.42 (0.80–7.33)	0.12
Body mass index (kg/m2)	1.19 (1.02–1.39)	0.028
Hypertension	2.25 (0.75–6.73)	0.15
Diabetes mellitus	1.19 (0.24–5.91)	0.83
Chronic obstructive pulmonary disease	1.00 (0.99–1.02)	0.22
Chronic heart failure	0.59 (0.18–1.95)	0.38
American Society of Anesthesiologists classification	1.73 (0.85–3.49)	0.13
Angiotensin-converting-enzyme inhibitors	0.91 (0.18–4.68)	0.91
Angiotensin receptor blockers	0.91 (0.27–3.02)	0.87
Atrial fibrillation	6.18 (1.71–22.3)	0.0054
Emergency	2.48 (0.76–8.10)	0.13
Operative time (hour)	1.31 (0.76–2.27)	0.33
Cardiopulmonary bypass time (min)	1.31 (0.76–2.27)	0.55
Estimated blood loss (/100 mL)	1.16 (1.03–1.30)	0.011
Urinary output	0.93 (0.85–1.01)	0.068
Crystalloid (/500 mL)	0.77 (0.57–1.04)	0.084
Hespander® (/500 mL)	2.05 (0.45–9.25)	0.35
Salinhes® (/500 mL)	0.53 (0.01–34.5)	0.77
Voluven® (/500 mL)	0.88 (0.31–2.53)	0.81
Red blood cells (/unit)	1.08 (0.89–1.30)	0.44
Fresh frozen plasma (/unit)	0.88 (0.79–0.99)	0.032
Platelet concentrates (/unit)	1.01 (0.95–1.07)	0.73
Autotransfusion (/unit)	0.78 (0.23–2.63)	0.68

A total of 481 patients in the HES group were matched to the 962 patients in the control group using propensity scoring. Preoperative and intraoperative patient characteristics were similar in the two groups. (Table 5).

**Table 5:** Propensity-matched comparison of study patients who did or did not receive HES before or after cardiopulmonary bypass

	HES	Control	Standardized difference
Preoperative parameters			
Number of patients	481	962	
Age (years)	65.9 ± 13.2	66.2 ± 12.2	2.74
Male gender	292 (60.7%)	581 (60.4%)	0.65
Body mass index (kg/m^2^)	23.1 ± 3.8	22.9 ± 3.2	0.49
Preoperative creatinine (mg/dL)	0.92 ± 0.40	0.93 ± 0.40	2.17
Emergency	180 (37.4%)	369 (38.4%)	−1.99
Preoperative comorbidities			
Hypertension	289 (60.0%)	587 (61.0%)	−1.96
Diabetes mellitus	68 (14.1%)	142 (14.8%)	−2.52
Chronic obstructive pulmonary disease	12 (2.5%)	23 (2.4%)	2.18
Chronic heart failure	192 (39.9%)	383 (39.8%)	0.22
Atrial fibrillation	113 (23.5%)	232 (24.1%)	−1.72
American Society of Anesthesiologists Classification			
1	10 (2.1%)	20 (2.1%)	
2	296 (61.5%)	553 (57.5%)	
3	143 (29.7%)	314 (32.6%)	
4	26 (5.4%)	72 (7.5%)	
5	6 (1.2%)	3 (0.3%)	
Preoperative medication			
Angiotensin-converting-enzyme inhibitors	82 (17.0%)	170 (17.7%)	−2.17
Angiotensin II receptor blockers	168 (34.9%)	341 (35.4%)	−1.14
Intraoperative parameters			
HES (mL)	692 ± 387	–	
Hespander® (mL)	14 ± 104		
Salinhes® (mL)	627 ± 432		
Voluven® (mL)	50 ± 186		
Crystalloids (mL)	2524 ± 1627	2553 ± 1129	2.06
Red blood cells (mL)	457 ± 959	511 ± 858	5.84
Fresh frozen plasma (mL)	392 ± 628	440 ± 653	7.60
Platelet concentrates (mL)	102 ± 193	112 ± 192	4.99
Estimated blood loss (mL)	669 ± 656	715 ± 681	6.82
Urinary outputs (mL)	1387 ± 884	1391 ± 846	0.48
Operative time (min)	356 ± 124	364 ± 116	7.16
Cardiopulmonary bypass time (min)	170 ± 67	173 ± 61	5.53
Longest interval of mABP < 65 (min)	66 ± 54	67 ± 53	1.92
Total mABP < 65 duration (min)	210 ± 107	215 ± 114	5.09
Operation procedures			
On-pump CABG	8 (1.7%)	14 (1.5%)	
Valve surgeries	261 (54.3%)	489 (50.8%)	
Valve + CABG	34 (7.1%)	93 (9.7%)	
Major aortic surgeries	152 (31.6%)	316 (32.8%)	
Others	26 (5.4%)	50 (5.2%)	

Data are expressed as means with standard deviation or percentages

*HES* hydroxyethyl starch, *mABP* mean atrial blood pressure, *CABG* coronary artery bypass graft

Outcomes are shown in Table 6. The incidence of AKI within 7 days after surgery in the HES group was not significantly different from the control group (26.6% vs. 26.9%, *p* = 0.95). The need for postoperative RRT (5.0% in the HES group and 4.5% in the control group, *p* = 0.757) and hospital mortality (3.3% in the HES group and 2.8% in the control group, *p* = 0.702) were not significantly different between the two groups. ICU length of stay was longer (3 (3–5) days vs. 3 (3–4) days, *p* = 0.0301) in the HES group than in the control group.

**Table 6:** Propensity-matched comparison outcomes in patients who did or did not receive HES before or after cardiopulmonary bypass

	HES (*n* = 481)	Control (*n* = 962)	*p* value
AKI incidents	128 (26.6%)	259 (26.9%)	0.95
AKI stage			
1	81 (16.8%)	162 (16.8%)	
2	17 (3.5%)	42 (4.4%)	
3	30 (6.2%)	55 (5.7%)	
Peak creatinine (mg/dL)	0.94 (0.76–1.26)	0.95 (0.74–1.35)	0.878
Need for RRT	24 (5.0%)	43 (4.5%)	0.757
ICU length of stay (days)	3 (3–5)	3 (3–4)	0.0301
28-day hospital free days (days)	11 (5–14)	12 (5–15)	0.293
Hospital mortality	16 (3.3%)	27 (2.8%)	0.702

Data are expressed as medians with 25th to 75th percentiles or percentages

*HES* hydroxyethyl starch, *AKI* acute kidney injury, *RRT* renal replacement therapy, *ICU* intensive care unit

To investigate the dose-dependent effects of HES, we compared the outcomes between patients who received 1000 mL or more of HES and those who received less than 1000 mL of HES before or after CPB (Table 3). Peak serum creatinine levels (*p* = 0.038), need for RRT (*p* = 0.017), and in-hospital mortality (*p* = 0.017) were higher, and 28-day hospital-free days was less in patients receiving HES ≥ 1000 mL than in those receiving HES < 1000 mL. The mean cost of fluid administration for each patient in the HES group was 2443 ± 1136 yen (corresponding to 21.6 ± 10.0 US dollars), whereas the control group was 1214 ± 482 yen (corresponding to 10.7 ± 4.3 US dollars, with a conversion rate of 113.25 yen/dollar) (*p* < 0.001).

## Discussion

This study evaluated the effect of intraoperative HES administration on postoperative kidney function. Propensity matching was performed to adjust for selection biases, showing that HES administration is not associated with an increase in the incidence of postoperative AKI. However, compared to HES < 1000 mL, administration of HES ≥ 1000 mL may be associated with worse postoperative outcomes including significantly higher serum creatinine levels, greater need for RRT, higher in-hospital mortality, and lower 28-day hospital-free days.

Compared to previous studies examining the impact of HES administration on the incidence of postoperative AKI, the present study included a greater number of patients. Only a few studies have evaluated renal function after administration of HES 70/0.5 (Endo et al. 2012), a widely used solution in Japan. This study evaluated the effects of intraoperative administration of HES 70/0.5 as well as HES 130/0.4 on postoperative kidney function.

On a molecular level, HES is believed to cause kidney injury due to its high oncotic pressure, which can directly decrease glomerular filtration, resulting in interstitial inflammatory changes (Schortgen and Brochard 2009; Hüter et al. 2009). From a clinical perspective, the CHEST study (Myburgh et al. 2012) showed that administration of HES was related to an increase in the need for RRT, supporting previous studies. The 6S study (Perner et al. 2012) also supports the possibility of a deleterious effect of HES at the molecular level, showing a relationship between administration of HES and 90-day mortality and the need for RRT.

Vast majority (90.7%) of patients who received any type of HES were administered with HES 70/0.5 in the operating room in the present study, while all participants were administered with HES 130/0.7 in the ICU in the previous studies (Myburgh et al. 2012; Perner et al. 2012). Endo et al. (Endo et al. 2012) investigated the relationship between the intraoperative sole use of HES 70/0.5 in mixed surgical patients including cardiac surgery and the incidence of AKI, showing no correlation. In Endo’s study (Endo et al. 2012), lower molecular weight HES (70/0.5) might have led to less accumulation in the kidney with fewer patients developing AKI, while in the current study, a larger amount of HES administration (≥ 1000 ml) resulted in a greater incidence of needing RRT and higher peak serum creatinine levels (Table 3). Either additional administration of higher molecular weight HES (130/0.7) or the patient characteristics (i.e., cardiac surgical patients) might have affected the results.

In the present study, the use of HES had no effect on the total volume of fluid administered as shown in previous studies (Perner et al. 2012; Lagny et al. 2015). In a study by Lagny (Lagny et al. 2015), the cumulative fluid balance over 48 h was 1077 ± 1698 in the crystalloid group and 1563 ± 1844 in the HES groups, a significantly greater volume of fluid administered to the HES group. Based on the results showing no reduction in total fluid administration along with the potential adverse effects in patients receiving a higher amount of HES, there may be no clinical benefit when using this small amount of HES. Since the cost of the fluid administered to the HES group was approximately twice as much as fluid for the control group, it is difficult to rationalize the deliberate use of HES during cardiac surgery.

We acknowledge several limitations in the current study. First, this is a retrospective study, and unrecognized confounding factors could affect the results. To minimize the effect of confounding factors, we performed propensity-matched analysis including a wide range of preoperative and intraoperative variables associated with the use of HES. Second, since this is a single-center study, external validity may be limited. However, the inclusion of a large number of patients (approximately 2000) undergoing a wide range of operative procedures over a relatively long period (8 years) may partially overcome the limitation. Third, all patients were given 500 mL of 6% HES (0.7/0.5) as a priming volume for CPB, which could obscure potential differences between the groups. We intended to investigate the effects of HES solutions used before or after CPB, when anesthesiologists are in charge of the fluid and hemodynamic management. In the present study, the average of 719 mL of HES was administered in the HES group, while in previous studies, a similar dose was administered in the HES groups with mixed results (Ishihara 2014; Endo et al. 2012). The present study suggests a potential harm by the use of a larger amount of either HES solution (70/0.5 or 130/0.4), which may add another relevant information for anesthesiologists. Fourth, for data analysis, patients given either HES 70/0.5 or HES 130/0.4 were allocated to a single “HES-administered” group since the number of patients who received HES 130/0.4 was limited, which could affect the results of the analysis. Fifth, AKI may appear after the initial 7 days of ICU stays and may be missed if the data collection on AKI is limited to the initial 7 days. However, we focused solely on the effects of intraoperative use of HES and intended to exclude the postoperative factors potentially affecting renal function. A previous study examining the effects of intraoperative use of HES on postoperative renal function also had the identical study period (Endo et al. 2012).

According to this retrospective study, administration of HES to patients undergoing cardiothoracic surgery before or after CPB was not associated with the incidence of postoperative AKI. However, a larger amount of HES may be associated with worse clinical outcomes. A large-scale randomized controlled study will be needed to establish the efficacy and safety of intraoperative administration of HES.

## Data Availability

The datasets used and/or analyzed during the current study are available from the corresponding author on reasonable request.
